# Organ Homologies and Perianth Evolution in the *Dasymaschalon* Alliance (Annonaceae): Inner Petal Loss and Its Functional Consequences

**DOI:** 10.3389/fpls.2018.00174

**Published:** 2018-02-20

**Authors:** Xing Guo, Daniel C. Thomas, Richard M. K. Saunders

**Affiliations:** ^1^School of Biological Sciences, The University of Hong Kong, Hong Kong, Hong Kong; ^2^Singapore Botanic Gardens, Singapore, Singapore

**Keywords:** Annonaceae, inner petal loss, organ homology, perianth evolution, *Dasymaschalon* alliance

## Abstract

The *Dasymaschalon* alliance within the early divergent angiosperm family Annonaceae comprises c. 180 species in four genera (*Dasymaschalon, Desmos, Friesodielsia*, and *Monanthotaxis*). The alliance offers an excellent opportunity for investigating perianth evolution and functional adaptations because of the presence of different numbers of petal whorls and contrasting floral chamber morphologies. The absence of the inner petal whorl in *Dasymaschalon* renders it distinctive in the family: previous studies have suggested that its three outermost stamens might be homologous with the inner petals of the sister genus, *Friesodielsia*, reflecting a homeotic shift of floral organ identify from inner petals to stamens. To investigate this hypothesis and general perianth evolution in the alliance, we (i) compared the floral vascularization of selected *Dasymaschalon* and *Friesodielsia* species using paraffin serial sectioning, and (ii) mapped selected perianth characters of inferred functional significance onto a molecular phylogenetic framework of the *Dasymaschalon* alliance (46 accessions; five cpDNA, and two nrDNA markers). The results indicate that the vasculature of the outermost stamen whorl of *Dasymaschalon* does not fuse with the perianth cortical vascular system, but instead splits from the basal traces of the free stamen bundles, contradicting previous inferences of homology with the inner corolla whorl of other Annonaceae. The loss of the inner petal whorl in *Dasymaschalon* is less likely to be due to a homeotic mutation, and instead possibly involved either the loss of genes that are responsible for determining inner petals or else the expression failure of these genes. Optimizations of perianth characters indicate that the absence of the inner petal whorl and the connivence of outer petals during anthesis are synapomorphic for *Dasymaschalon*. Circadian trapping of pollinators is inferred either to be derived in the stem lineage of the *Dasymaschalon–Friesodielsia* clade, or else to have evolved in parallel in the *Dasymaschalon* and *Friesodielsia* lineages. Subsequent changes in the remaining petals of *Dasymaschalon* flowers (which do not fully separate during anthesis) are likely to have enabled perpetuation of the circadian trapping mechanism, lessening the adverse impacts of inner petal loss.

## Introduction

**FIGURE 1:**
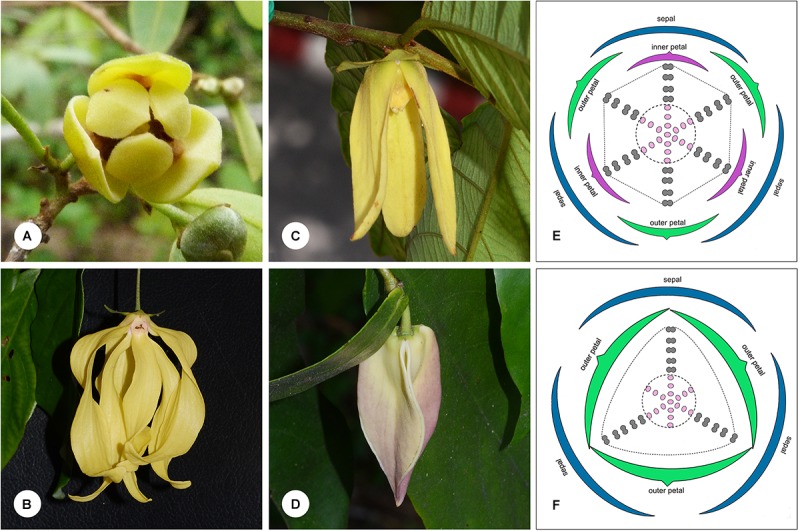
Floral morphology in the *Dasymaschalon* alliance. **(A)** *Monanthotaxis buchananii*, showing loosely coherent pollination chamber. **(B)** *Desmos chinensis*, with partially closed chamber formed by petals that are basally constricted. **(C)** *Friesodielsia desmoides*, with outer petals free spreading, inner petals apically connivent forming a closed pollination chamber. **(D)** *Dasymaschalon dasymaschalum*, with three outer petals apically connivent forming a closed pollination chamber. **(E)** Floral diagram of *Friesodielsia*. **(F)** Floral diagram of *Dasymaschalon*. Colors are used to differentiate floral organs; blue, sepal; green, outer petal; purple, inner petal; gray, stamen; pink, carpel. The ranks and total numbers of stamens and carpels are artificial in **(E,F)**, with six/three lines of stamens and carpels shown at the corners of the hexagonal/triangular floral meristem. — Photographs: **(A)** Warren McCleland; **(B–D)** Xing Guo.

The *Dasymaschalon* alliance (Annonaceae subfam. Annonoideae tribe Uvarieae; Chatrou et al., 2012; Guo et al., 2017b) comprises a group of four closely related paleotropical genera: *Dasymaschalon* (Hook. f. & Thomson) Dalla Torre & Harms, *Desmos* Lour., *Friesodielsia* Steenis and *Monanthotaxis* Baill. It is a large lineage, with c. 180 species (Guo et al., 2017a), widely distributed in tropical Africa and tropical Asia. The *Dasymaschalon* alliance is remarkably diverse in floral morphology (**Figure 1**), with different numbers of petals and contrasting floral pollination chamber morphologies, and hence represents a model for investigating perianth evolution and functional adaptations.

The genera *Desmos, Friesodielsia* and *Monanthotaxis* are largely uniform in their underlying floral ‘Bauplan’ and are similar to most other Annonaceae species: the flowers are trimerous with a whorl of sepals and two morphologically distinct whorls of petals. The three inner petals differ between the three genera, however, and are either freely spreading (*Monanthotaxis*: **Figure 1A**), basally constricted (*Desmos*: **Figure 1B**), or apically connivent (*Friesodielsia*: **Figure 1C**), forming contrasting pollination chambers in combination with the three outer petals in the latter two genera. Unlike most species of the alliance and the family as a whole, the flowers of *Dasymaschalon* only have one whorl of three petals (**Figure 1D**), which are regarded as homologous with outer petals of the other three genera in the alliance due to their alternating position relative to the sepals (Saunders, 2010; Wang et al., 2012). Obvious differences that can distinguish *Dasymaschalon* from its close relatives *Desmos* and *Friesodielsia* are also observed in the number of stamens in the outermost whorl: *Desmos* and *Friesodielsia* flowers have six outer stamens located directly in front of the inner or outer petals (**Figure 1E**), whereas *Dasymaschalon* flowers only have three outer stamens located at the corners of the triangular floral meristem (**Figure 1F**).

The evolutionary changes in floral morphology in *Dasymaschalon* can be explained by reference to possible organ homologies: the three outermost stamens in *Dasymaschalon* might be homologous with the three inner petals in *Desmos* and *Friesodielsia*, a suggestion that corresponds well with the hypothesis of a homeotic shift in floral organ identity (Saunders, 2010; Wang et al., 2012) from outer petals to inner petals and from inner petals to stamens. Alternatively, the three outermost stamens of *Dasymaschalon* might be typical stamens and the three inner petals may have been lost, probably due to the loss or suppression of relevant genes controlling inner petal development. Detailed comparative analysis of floral structure in *Dasymaschalon* and its close relatives is needed to explore organ homologies.

Ancestral character reconstruction by mapping of morphological traits onto a phylogenetic tree is an invaluable approach to enable identification of synapomorphies and the inference of evolutionary patterns and trends. Wang et al. (2012) performed ancestral character state reconstructions for four reproductive characters within the *Dasymaschalon* alliance using a phylogenetic tree based on a concatenated dataset from five chloroplast regions (*matK, ndhF, rbcL, psbA-trnH*, and *trnL-F*). The results suggested that *Dasymaschalon* is not monophyletic and the three-petalled corolla characteristic of the genus was consequently interpreted to have evolved independently twice within the alliance. The study could not determine whether *Dasymaschalon* is paraphyletic or polyphyletic, however, due to a basal polytomy in the *Desmos–Dasymaschalon–Friesodielsia* lineage. Subsequent molecular phylogenies (Guo et al., 2017a) significantly increased the sampling of taxa and DNA regions, using a combined dataset consisting of chloroplast and nuclear DNA sequences, enabling new insights into the relationships within the *Dasymaschalon* alliance. This research resolved intergeneric relationships, with *Dasymaschalon* shown to be most closely related to Asian representatives of *Friesodielsia* (with African species previously classified in this genus transferred to *Monanthotaxis*). A case of well-supported incongruence between the chloroplast and nuclear DNA phylogenies was furthermore identified in *Dasymaschalon*, suggesting a likely ancient hybridization between the *Dasymaschalon* and Asian *Friesodielsia* lineages (Guo et al., 2017a). The availability of well-resolved phylogenies and the increasingly comprehensive understanding of species diversification provides an excellent opportunity for reassessing floral diversity and perianth evolution in the *Dasymaschalon* alliance.

The aims of this study are therefore to assess putative organ homologies in *Dasymaschalon*, and to investigate the evolution of functionally important floral characters in *Dasymaschalon* and allied genera. To achieve these aims, comparative studies of floral vascularization are undertaken for selected species from *Dasymaschalon* and its sister genus *Friesodielsia* using paraffin serial sectioning. In addition, selected perianth characters of functional significance are mapped onto the phylogeny (based on seven chloroplast and nuclear DNA markers) to trace the evolutionary pattern, identify synapomorphies, and reveal potential morphological adaptation with regard to ecological function.

## Materials and Methods

### Paraffin Serial Sectioning

Mature flowers of *Dasymaschalon trichophorum* Merr. (*X. Guo 20130517-1*, Hainan, China [HKU]) and *Friesodielsia desmoides* (Craib) Steenis (*X. Guo 20130630-1*, Thailand [HKU]) were collected and preserved in freshly prepared formalin-acetic acid-alcohol (FAA; 1:1:18 mixture of 40% formalin, glacial acetic acid and 70% ethanol) for 24 h, and then transferred to 70% ethanol for long-term storage at room temperature. The specimens were subsequently dehydrated using a tertiary butyl alcohol (TBA) series (Ruzin, 1999) prior to embedding in paraffin wax and sectioning (12 μm thickness) using a rotary microtome. Serial sections were sequentially mounted onto clean slides using gelatin, dried overnight and dewaxed using Histochoice Clearing Agent (Sigma–Aldrich). The slides were then stained with Safranin O and Fast Green, mounted with DPX (Ruzin, 1999), and photographed using a Nikon 80i imaging system (voucher slides deposited in HKU herbarium).

### Ancestral Character State Reconstructions

A Bayesian molecular phylogeny of the *Dasymaschalon* alliance (Guo et al., 2017a) was used to reconstruct ancestral character states. Four perianth characters of diagnostic and functional importance were selected. Character states were scored based on species descriptions and direct observations from living and herbarium materials. The following characters and character states were studied: (i) number of petal whorls: 0 = one whorl; 1 = two whorls; (ii) pollination chamber type: 0 = pollination chamber absent, with petals spreading or loosely coherent (Type I); 1 = partially closed pollination chamber formed by basally constricted petals, with an apical aperture and three basal apertures (Type II); 2 = tightly closed pollination chamber formed by connivent inner petals, with three basal apertures periodically blocked by outer petals (Type III); 3 = tightly closed pollination chamber formed by connivent outer petals (Type IV); (iii) connivence of outer petals when immature: 0 = free; 1 = connivent; and (iv) opening of outer petals during anthesis: 0 = becoming fully open; 1 = remaining apically connivent. The taxon-character matrix for the reconstructions is given in **Supplementary Table S1**, with variation in floral chambers among the different genera illustrated in **Figure 1**.

Ancestral character state reconstructions were performed in Mesquite v.3.2 (Maddison and Maddison, 2017) by parsimony and likelihood methods using MCMC trees after initial 50% burn-in as input tree file. A 50% majority-rule consensus tree was summarized from post-burn-in trees and used as the topology for mapping the reconstructions. As the analyses primarily focused on *Dasymaschalon* and its close relatives *Desmos* and *Friesodielsia*, samples of *Monanthotaxis*, as well as phylogenetically more distant outgroups, were pruned from the tree. The final 46-accession phylogeny included 10 *Desmos* accessions, 18 *Friesodielsia* accessions, and 15 *Dasymaschalon* accessions, with single species representatives of *Monanthotaxis, Sphaerocoryne*, and *Toussaintia* as outgroups.

For the parsimony optimizations, character state changes were treated as unordered. The option “Trace over trees” was used to account for phylogenetic uncertainty. Optimizations of each character across the input trees were summarized at each node using the “Count Trees with Uniquely Best States” option, with reconstructions regarded as equivocal when two or more states were estimated as equally parsimonious for a particular node.

For the likelihood approach, the Markov k-state 1 parameter (Mk1) model (Lewis, 2001) was selected for ancestral character state reconstructions: character state changes are regarded as equally probable under this model, and the only parameter is the rate of change. Likelihood reconstruction results were summarized at each node using the “Average Frequencies across Trees” option, which estimates the average likelihood of each state across the input trees.

## Results

**FIGURE 2:**
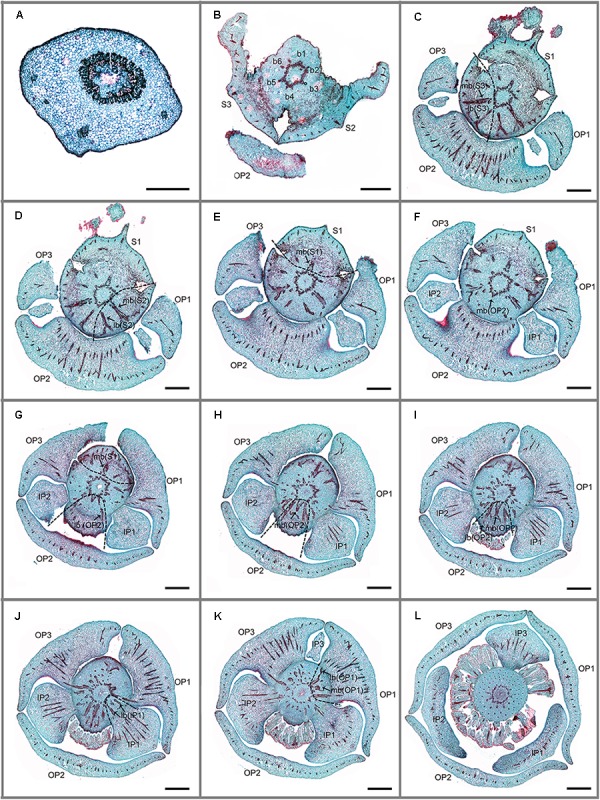
Transverse sections showing the floral vasculature of *Friesodielsia desmoides*. **(A–L)** are shown in sequence from base to apex. **(A)** Section through pedicel, showing stele. **(B)** Base of receptacle, showing six groups of vascular bundles. **(C–F)** Position where the sepals and outer petals are connected to receptacle, showing their median bundles and lateral bundles. **(G–I)** Position where sepals and outer petals are connected to receptacle, showing their median bundles and lateral bundles. **(J)** Position where inner petals are connected to receptacle, showing their median bundles and lateral bundles. **(K)** Vascular bundles leading to outer petal 1. **(L)** Vascular bundles of stamens. Floral organs (S, sepal; OP, outer petal; IP, inner petal); vascular bundles (b, bundle; lb, lateral bundle; mb, median bundle); numbers used to differentiate between organs from the same whorl. Scale bars: **(A)** = 0.5 mm; **(B–L)** = 1 mm.

**FIGURE 3:**
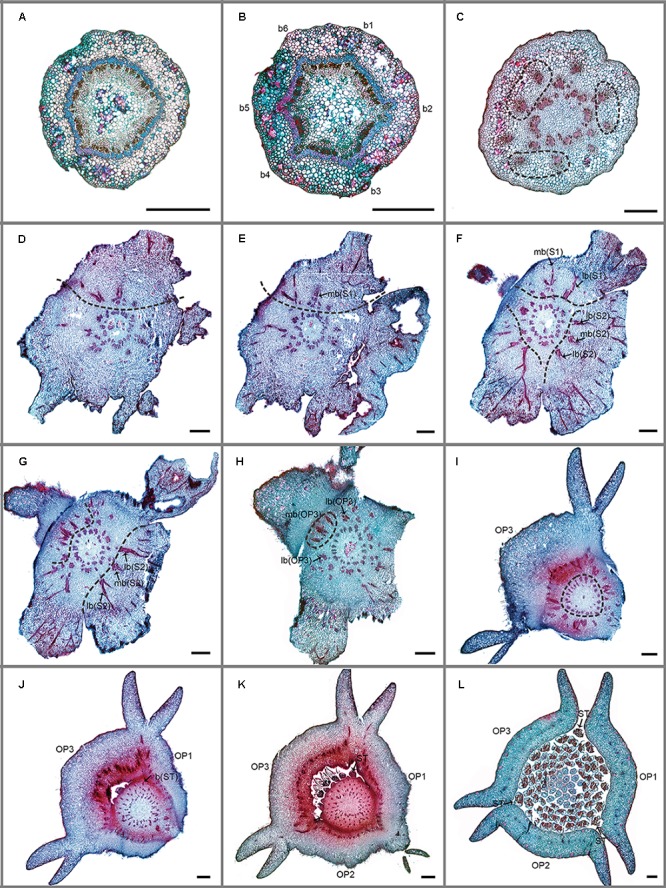
Transverse sections showing the floral vasculature of *Dasymaschalon trichophorum*. **(A–L)** are shown in sequence from base to apex. **(A)** Section through pedicel, showing stele. **(B)** Base of receptacle, showing six groups of vascular bundles. **(C)** Three pairs of lateral sepal bundles. **(D–F)** Position where sepals are connected to receptacle, showing their median bundles and lateral bundles. **(G,H)** Position where sepals and outer petals are connected to receptacle, showing their median bundles and lateral bundles. **(I–K)** Top of receptacle, showing vascular bundles leading to stamens. **(L)** Section through flower, above receptacle, showing positions of carpels, stamens relative to sepals and outer petals. Floral organs (S, sepal; OP, outer petal; IP, inner petal; ST, outermost stamen); vascular bundles (b, bundle; lb, lateral bundle; mb, median bundle); numbers used to differentiate between organs from the same whorl. Scale bars = 0.5 mm.

**FIGURE 4:**
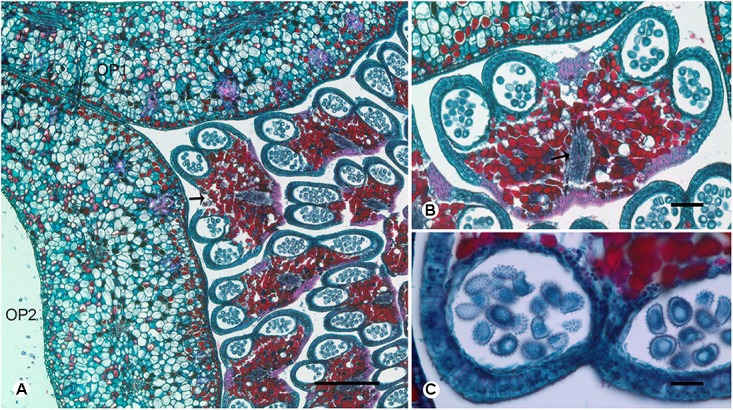
Transverse sections showing the stamen vasculature of *Dasymaschalon trichophorum*. **(A)** Section showing outermost stamen (indicated by arrow) at corner of adjacent outer petals. **(B)** Section through outermost stamen, showing four pollen sacs and vascular bundle (indicated by arrow). **(C)** Pollen grains of outermost stamen. OP, outer petal; numbers used to differentiate between organs from the same whorl. Scale bars: **(A,B)** = 0.5 mm; **(C)** = 1 mm.

**FIGURE 5:**
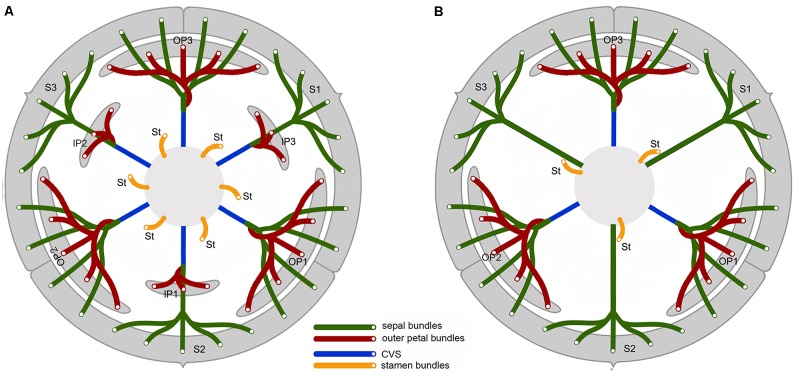
Vascular diagram of *Friesodielsia desmoides* **(A)** and *Dasymaschalon trichophorum* **(B)**. Abbreviations, with numbers used to differentiate between organs from the same whorl; CVS, cortical vascular system; IP, inner petal; OP, outer petal; S, sepal; St, stamen (indicated for outmost stamen whorl only).

Serial sections of *Friesodielsia desmoides* and *Dasymaschalon trichophorum* are illustrated in **Figures 2–4**. The vascular supply to each perianth organ is represented diagrammatically in **Figure 5**.

*Friesodielsia desmoides* possesses a vascular anatomy that is typical of the Annonaceae, comprising a perianth cortical vascular system (CVS, sensu Deroin, 1989, 1999) with three whorls of vascular traces. The sepals each possess three basal groups of vascular bundles (one median and two lateral bundles) whereas each petal (inner and outer) is supplied by a single basal group. The stele in the pedicel (**Figure 2A**) diverges into six groups of vascular bundles at the base of the receptacle (labeled b1–6 in **Figure 2B**): three of these bundle clusters (b1, b3, and b5 in **Figure 2B**) fuse with the median bundle of the sepals (mb in **Figures 2C–E**) and the vasculature supplying the inner petals (**Figure 2J**); the other three groups (b2, b4, and b6 in **Figure 2B**) connect with the vasculature of the outer petals (**Figures 2F,G–I,K**) and two lateral bundles feeding adjacent sepals (lb in **Figures 2C,D**).

The floral vascular anatomy of *D. trichophorum* is similar to that of *F. desmoides*: the cortical stele in the pedicel (**Figure 3A**) diverges to form six clusters of vascular bundles (b1–6 in **Figure 3B**); the vasculature of the first perianth whorl consists of three clusters of vascular bundles, each leading to one median and two lateral traces (**Figures 3D–G**). In contrast with the vasculature of *Friesodielsia* and most other Annonaceae with a tripartite perianth, however, *D. trichophorum* flowers possess two whorls of vascular traces (**Figures 3, 5B**), with the vasculature supplying the third perianth whorl in *F. desmoides* absent in *D. trichophorum*.

**FIGURE 6:**
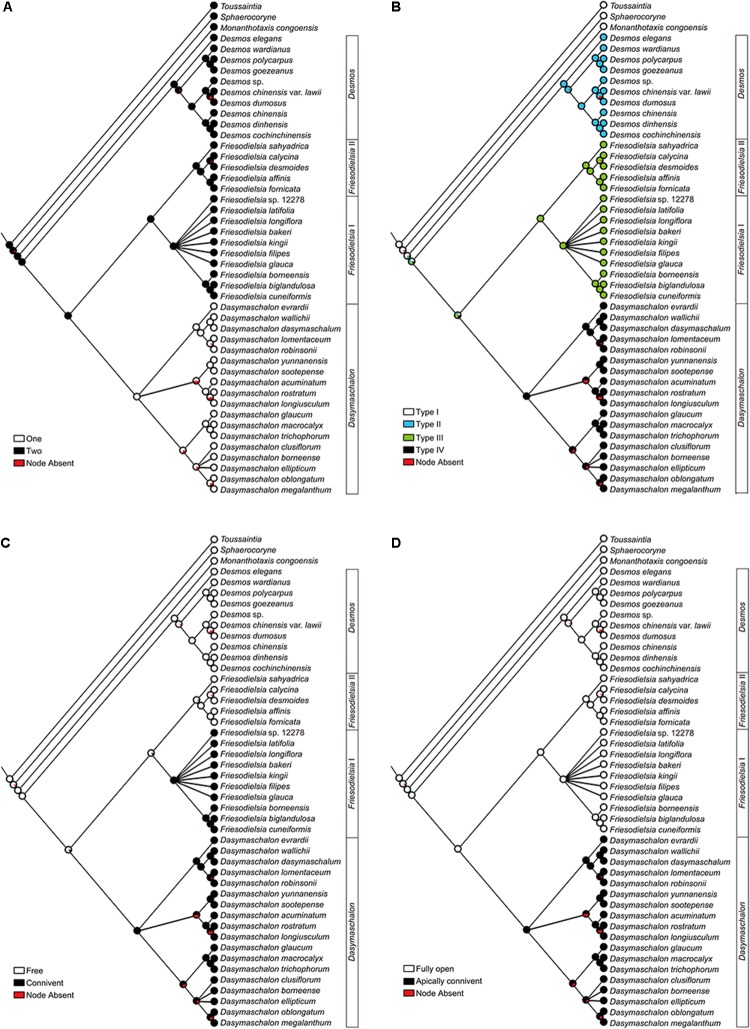
Likelihood ancestral character reconstructions for the *Desmos–Dasymaschalon–Friesodielsia* clade. **(A)** Number of petal whorls. **(B)** Pollination chamber type. **(C)** Connivence of outer petals in flower buds. **(D)** Opening of outer petals during anthesis. Character reconstructions across 10,000 Bayesian input trees are summarized and mapped on the 50% majority-rule consensus tree based on concatenated dataset from seven chloroplast and nuclear DNA regions. Pie charts at each node show the ML mapping results for the ancestral state and the percentage of node absence in the input trees.

The results of the ancestral character reconstructions are presented in **Figure 6** and **Supplementary Figure S1**. The evolutionary patterns for each character are highly congruent, irrespective of whether parsimony and likelihood approaches are used.

The genus *Dasymaschalon* is shown to be strongly supported by three synapomorphies: character (i), one whorl of petals; character (ii), the tightly closed pollination chamber formed by connivent outer petals; and character (iv), outer petals that remain apically connivent during anthesis. Character (iii), connivent outer petals in the flower bud, appears to have evolved independently twice in the lineage: in addition to its evolution in *Dasymaschalon*, it is also apparent in the *Friesodielsia* I clade.

## Discussion

### Organ Homologies

The vasculature of the second perianth whorl in *Dasymaschalon trichophorum* is clearly connected to the lateral bundles of the adjacent parts of the first perianth whorl (**Figure 5B**). The vascular traces leading to various perianth organs are intimately connected, collectively forming a perianth cortical vascular system (CVS) that correspond to the outer and middle whorls widely reported in most other Annonaceae flowers (Deroin, 1989, 1999; Deroin and Le Thomas, 1989; Xue and Saunders, 2013). The two perianth whorls in *Dasymaschalon* flowers are therefore interpreted here as homologues of the calyx and outer corolla whorl observed in the sister genus *Friesodielsia* and other Annonaceae (**Figure 5**).

Cases of reduction or increase in the number of whorls are rare within the Annonaceae, although two clear examples, *Fenerivia heteropetala* Diels and *Dasymaschalon*, were highlighted in a review by Saunders (2010). *Fenerivia heteropetala* flowers possess three highly reduced sepals and 12 petals, comprising: three ovate outer petals; three linear bracteopetals, homologous with inner petals of other Annonaceae species; and six linear andropetals, derived from the outermost stamens (Deroin, 2007; Saunders, 2010). This has been interpreted as the possible result of disruptions to the homeotic control of floral organ identity during development, with a centrifugal shift so that the outermost whorl of six stamens develop as inner petals (Saunders, 2010; Xue and Saunders, 2013).

Conversely, a centripetal change in the homeotic control of floral organ identity has been hypothesized in *Dasymaschalon* (Saunders, 2010). The absence of inner petals and the reduction in the number of outermost stamens can be explained by reference to putative organ homologies, with the three outermost stamens in *Dasymaschalon* possible homologues of the three inner petals in the related genera *Desmos* and *Friesodielsia.* If this interpretation of organ homology is correct several structural features are expected, including: (1) fusion of the vascular traces of the outermost stamens with the CVS bundles; (2) three vascular bundles supplying the outermost stamens, similar to that observed for inner petals in contrast with typical stamens, which have a single trace; and possibly also (3) sterility of the outermost stamens (i.e., staminodes that lack functional pollen). Serial sections of *D. trichophorum* reveal that the vasculature of the outermost stamen whorl does not fuse with the CVS, but instead splits from the basal traces of the free stamen bundles (**Figures 3I–K**), providing no evidence for homology with the inner corolla whorl of *Friesodielsia.* The outmost stamens have a single vascular trace (**Figures 4A,B**) and possess four pollen sacs (**Figures 4A,B**) with fully developed pollen grains that are likely to be functional (**Figure 4C**). These features do not support the interpretation that the outermost stamens of *Dasymaschalon* are homologous with the inner petals of *Friesodielsia*.

According to the classic ABC model (Bowman et al., 1991; Coen and Meyerowitz, 1991) and modified ABCDE model (Rijpkema et al., 2010, and references therein) of flower development, floral organ identity in eudicots can be determined by several classes of homeotic genes (essentially MADS-box genes). The ABC model is also likely to be applicable to the family since it has been tentatively identified in other members of the Annonaceae (*Asimina longifolia* Kral: Kim et al., 2005): B class genes AP3 and PI were expressed in petals and stamens, and not expressed or only weakly expressed in sepals. In *Dasymaschalon*, however, the inner petal whorl is likely lost and hence a homeotic conversion in identity is probably not involved. Instead, the likely cause might be the loss of genes that are responsible for determining inner petals or expression failure of these genes. *PETAL LOSS* genes may influence a petal initiation signal in the *Arabidopsis* flower (Griffith et al., 1999). In the buttercup family (Ranunculaceae), disruption of the petal identity gene *APETALA3-3* is highly correlated with loss of petals (Zhang et al., 2013), and the underlying genetic mechanisms may be complex, with disruption of *AP3-3* being either cause or effect (organ identity function likely not involved). The genetic control of floral morphological changes of the *Dasymaschalon* alliance is largely unknown, and further study involving flower development gene expression approaches (transcriptomes, RT-PCR, and *in situ* hybridization) is needed to investigate whether similar genes are likely associated with the inner petal loss in *Dasymaschalon*.

### Perianth Evolution

#### Occurrence of an Inner Petal Whorl

Our character optimizations (**Figure 6A** and **Supplementary Figure S1A**) suggest that the ancestor of the *Dasymaschalon* alliance possessed flowers with inner and outer petal whorls, with the loss of the inner whorl synapomorphic for *Dasymaschalon*.

The previous phylogenetic study (Wang et al., 2012) that demonstrated non-monophyly of *Dasymaschalon* was based solely on chloroplast DNA sequence data, and hence provided no opportunity for assessing putative reticulate evolution; the authors accordingly inferred that the derived three-petalled condition was likely to have evolved independently in the small *D. filipes*–*longiflorum*–*tibetense* clade and in the main *Dasymaschalon* clade comprising all other species in the genus. Subsequent phylogenetic analyses (Guo et al., 2017a) based on nuclear and chloroplast DNA sequence data from unlinked genomes, however, suggested that ancient hybridization had likely occurred between ancestors of the *Dasymaschalon* and *Friesodielsia* lineages, resulting in incongruent positions of a clade of three species (*D. filipes, D. longiflorum* and *D. tibetense*) in cpDNA and rDNA phylogenies. The loss of the inner petal whorl is likely to have occurred only once in the alliance, in the ancestor of the entire *Dasymaschalon* clade. We infer that the maternal parent of the hybridization event might belong to *Friesodielsia* lineage since *D. filipes*–*longiflorum*–*tibetense* clade is more closely related to *Friesodielsia* in the cpDNA tree. The occurrence of this trait in the species *D. filipes, D. longiflorum*, and *D. tibetense* is likely to be due to inheritance from the paternal parent (an ancestor of the *Dasymaschalon* clade).

#### Types of Floral Chambers

Genera within the *Dasymaschalon* alliance possess a diversity of pollination chamber types (**Figure 1**). Our optimizations (**Figure 6B** and **Supplementary Figure S1B**) suggest that the absence of a pollination chamber in *Monanthotaxis* (Type I; **Figure 1A**) is the ancestral state; this is consistent with the general perianth evolutionary pattern of Annonaceae reconstructed by Saunders (2010) and the morphology of *Futabanthus asamigaensis* Takahashi et al. (2008) the earliest known fossil flower of this family, dating from the Late Cretaceous of Japan. Floral chambers are invariably derived in the *Desmos*–*Dasymaschalon*–*Friesodielsia* clade with each chamber type synapomorphic for the corresponding genus.

The chamber in *Desmos* flowers is only partially enclosed, and is formed by the basal constriction of petals around the carpels and stamens (Type II; **Figure 1B**). This type also occurs in *Artabotrys*, the *Cananga*–*Cyathocalyx*–*Drepananthus* clade and *Pseudartabotrys* (‘Type II’ sensu Saunders, 2010), suggesting extensive parallel evolution of pollination chambers in the Annonaceae. Floral chambers in the Annonaceae are regarded as an adaptation to enhance pollination by small beetles (Saunders, 2010), providing protection for reproductive organs, providing shelter and a copulation site for pollinators, encouraging pollinator mobility by enabling microenvironments with elevated temperatures within the chamber, and filtering out larger insects with a body size that exceed the diameter of the apertures. The considerable homoplasy in floral chambers in the Annonaceae presumably reflects a strong functional significance associated with pollination mechanisms (Saunders, 2010).

**FIGURE 7:**
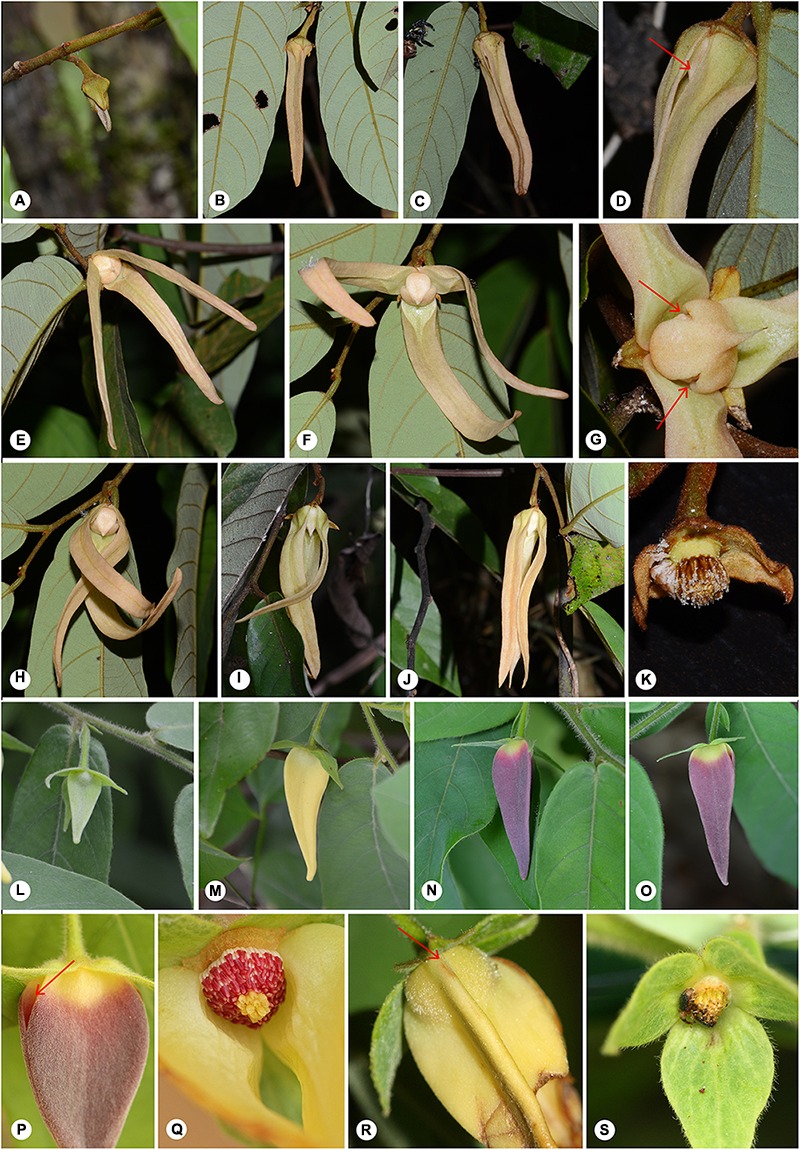
Flower-level phenological changes in *Friesodielsia borneensis* **(A–K)** and *Dasymaschalon trichophorum* **(L–S)**. *Friesodielsia borneensis*: **(A)** Early bud. **(B)** Petal elongation. **(C,D)** Petal separation, from petal base toward apex. **(E,F)** Outer petals move up and three apertures open. **(G)** Apertures fully open (arrowed). **(H–J)** Outer petals move down and three apertures closed. **(K)** Petals abscised. *Dasymaschalon trichophorum*: **(L)** Early bud. **(M)** Petal elongation. **(N)** Mature stage, with three petals connivent. **(O)** Petal separation at the base and three apertures start to open. **(P)** Apertures fully open, abaxial view (arrowed). **(Q)** Apertures fully open, adaxial view. **(R)** Three apertures closed (arrowed). **(S)** Petals abscised. — Photographs: **(A–O)** Xing Guo. **(P–S)** Chun Chiu Pang.

*Friesodielsia* flowers possess a tightly enclosed pollination chamber formed by connivence of the inner petals, with three apertures at the base of contiguous inner petals periodically blocked by the three outer petals (Type III). In contrast, *Dasymaschalon* flowers, which lack inner petals, have an enclosed pollination chamber formed by connivent outer petals (Type IV). Despite the evidently heterologous origin of floral chambers in *Dasymaschalon* and *Friesodielsia*, pollination ecology data (Lau et al., 2017) indicate that both genera possess time-dependent (circadian) trapping of pollinators, controlled by the movement of petals (see flower-level phenological changes in **Figure 7**: *Friesodielsia borneensis*, A–K and *Dasymaschalon trichophorum*, L–S): the chamber apertures are exposed during the pre-receptive period (in *Friesodielsia*, because the outer petals reflex to expose the apertures between the inner petals; or in *Dasymaschalon* because of lateral petal growth); beetles are attracted to the flowers by olfactory cues at the beginning of the pistillate phase in the early morning (Day 1 of anthesis), but are later trapped within flowers by subsequent petal movement; the flowers then remain completely closed during the interim and staminate phases, subsequently abscising to release the beetles at the end of the staminate phase (early morning of Day 2).

Lau et al. (2017) discovered that the opening and closure of the floral traps are very precisely timed in relation with pollinator activity patterns. This circadian trapping endows a major selective advantage by enabling the utilization of a broader range of potential pollinators, including those with circadian rhythms that are both unimodal (with a single daily activity peak) and bimodal (with twice-daily peaks). In *Desmos*, which is sister to the *Dasymaschalon–Friesodielsia* clade, the flowers lack this complex circadian trapping mechanism, but otherwise share several similarities in pollination system with *Dasymaschalon* and *Friesodielsia* (e.g., abbreviated anthesis of 23–27 h duration, small beetles as effective pollinators, and pistillate/staminate-phase floral synchrony: Pang and Saunders, 2015; Lau et al., 2017). *Desmos* flowers therefore face limitations in utilizing pollinators with a bimodal circadian activity because the apertures between petals are open throughout anthesis and cannot prevent the departure of pollinators during the second activity peak.

#### Connivence of Outer Petals

The three petals in *Dasymaschalon* flowers (homologues of outer petals in *Friesodielsia*) are firmly connivent in young flower buds (**Figures 7L–N**). The outer petals in *Friesodielsia* differ in the two main clades retrieved in phylogenetic reconstructions: species in the *Friesodielsia* I clade closely resemble the petals of *Dasymaschalon*, whereas species in the *Friesodielsia* II clade have flower buds with freely spreading outer petals (**Figure 6C** and **Supplementary Figure S1C**). Our likelihood ancestral character state reconstructions (**Figure 6C**) indicate that the separate, non-connivent outer petal condition is plesiomorphic, whereas connivence of outer petals has been achieved independently in the *Friesodielsia* I and *Dasymaschalon* clades, although parsimony reconstructions are equivocal (**Supplementary Figure S1C**). It is likely that the firmly connivent outer petals in immature flowers provide protection for the inner petals and reproductive organs: *D. trichophorum* petals have been observed to be consumed by *Pathysa antiphates* caterpillars (C. C. Pang, personal communication), and damage after oviposition by beetle pollinators is common in *F. borneensis* (Guo, personal observation).

The connivent outer petals characteristic of species in the *Friesodielsia* I clade (**Figures 7A,B**) gradually separate and reflex as flowers mature (Guo et al., 2017a), exposing small basal apertures between the inner petals (**Figures 7C–G**). The petals of *Dasymaschalon* closely resemble the outer petals of species in the *Friesodielsia* I clade, which have a very similar size, shape and structure, and also remain connivent in immature flowers. *Dasymaschalon* petals only separate at the base, however, with the apical region remaining connivent throughout the sexually functional period until the end of the staminate phase (Pang and Saunders, 2014; Guo, personal observation). This characteristic occurs in almost all species of *Dasymaschalon* (with the exception of *D. longiflorum*), suggesting that it is synapomorphic for the genus (**Figure 6C** and **Supplementary Figure S1C**).

The strength of the contact between the apically connivent petals varies among different species of *Dasymaschalon*, although the petals are firmly connivent in most species (e.g., *D. dasymaschalum* (Blume) I.M.Turner: **Figure 1D**; *D. trichophorum*: **Figures 7L–R**). *Dasymaschalon acuminatum* Jing Wang & R.M.K.Saunders, for example, has petals that are relatively firmly convergent at the apex but much more loosely associated toward the base. In *D. sootepense* Craib, the three petals are essentially separate or only weakly connivent over a small area at the apex. These variations in the strength of contact of petals presumably reflect an accumulation of smaller changes in response to selective pressures, which may be associated with pollination mechanisms.

### Adaptive Change in Outer Petals of *Dasymaschalon*

Most Annonaceae species possess partially enclosed pollination chambers with apertures that remain open throughout anthesis so that pollinators can freely enter or depart from the flowers during any phenological stage (Saunders, 2010). Tightly enclosed pollination chambers associated with circadian trapping are rare in the family, only having been reported in *Dasymaschalon* (Pang and Saunders, 2014; Lau et al., 2017), *Friesodielsia* (Lau et al., 2017), *Goniothalamus* (Lau et al., 2016, 2017) and possibly also *Artabotrys* (Lau et al., 2017).

*Dasymaschalon* is distinctive amongst Annonaceae species reported to show circadian trapping: the outer petals of *Friesodielsia* (**Figures 7A–K**) and *Goniothalamus* species periodically reflex during anthesis to control the opening and closing of apertures, whereas *Dasymaschalon* petals remain firmly connivent throughout the anthesis, with closure of apertures due to only minimal lateral petal expansion (**Figures 7L,S**). Our reconstructions of ancestral character states (**Figures 6A,D** and **Supplementary Figures S1A,D**) indicate that loss of inner petals in *Dasymaschalon* may be associated with evolution of the connivence of the remaining petals during sexually functional stages. If the petals of *Dasymaschalon* were to reflex as in *Friesodielsia*, the pollination chamber would lose its ability to prevent beetles from leaving before pollen release. As discussed above, connivence of outer petals during sexually functional stages in *Dasymaschalon* varies in the degree of contact between adjacent petals, ranging from firmly convergent to only weakly connivent over a small area at the apex. The accumulation of small changes presumably provides further evidence for adaptation in response to selective pressures.

Circadian trapping likely evolved twice in the *Dasymaschalon* alliance, and multiple times independently in Annonaceae (tribe Annoneae: *Goniothalamus*; tribe Uvarieae: *Dasymaschalon, Friesodielsia*; and possibly tribe Xylopieae: *Artabotrys*), indicating considerable selective advantages of the mechanism. If circadian trapping already evolved in the stem lineage of the *Dasymaschalon–Friesodielsia* clade, the loss of inner petals in the *Dasymaschalon* ancestor would have disrupted it. The subsequent changes in *Dasymaschalon*, with petals that do not fully separate, were possibly adaptations lessening detrimental effects of inner petal loss resulting in re-establishment of the trapping mechanism and its associated selective advantages.

## Author Contributions

XG and RS conceived the study; XG undertook experiments and analyzed the data; and all authors wrote the manuscript.

## Conflict of Interest Statement

The authors declare that the research was conducted in the absence of any commercial or financial relationships that could be construed as a potential conflict of interest.

## Supplementary Material

FIGURE S1 Parsimony ancestral character reconstructions for the *Desmos–Dasymaschalon–Friesodielsia* clade. **(A)** Number of petal whorls. **(B)** Pollination chamber type. **(C)** Connivence of outer petals in flower buds. **(D)** Opening of outer petals during anthesis. Character reconstructions across 10,000 Bayesian input trees are summarized and mapped on the 50% majority-rule consensus tree based on concatenated dataset from seven chloroplast and nuclear DNA regions. Pie charts at each node show the MP mapping results for the ancestral state and the percentage of node absence in the input trees.

TABLE S1 Taxon-character data matrix used for ancestral character-state reconstructions. (i) number of petal whorls: 0 = one whorl; 1 = two whorls; (ii) pollination chamber type: 0 = pollination chamber absent, with petals spreading or loosely coherent; 1 = partially closed pollination chamber formed by basally constricted petals, with an apical aperture and three basal apertures; 2 = tightly closed pollination chamber formed by connivent inner petals, with three basal apertures periodically blocked by outer petals; 3 = tightly closed pollination chamber formed by connivent outer petals; (iii) connivence of outer petals when immature: 0 = free; 1 = connivent; (iv) opening of outer petals during anthesis: 0 = becoming fully open; 1 = remaining apically connivent.
