# Supplementary figures and images for: Organ Homologies and Perianth Evolution in the Dasymaschalon Alliance (Annonaceae): Inner Petal Loss and Its Functional Consequences
Source: Front Plant Sci. 2018 Feb 20;9:174. doi: 10.3389/fpls.2018.00174 (PMC5826315; doi:10.3389/fpls.2018.00174)

# SUPPLEMENTARY FIGURE S1

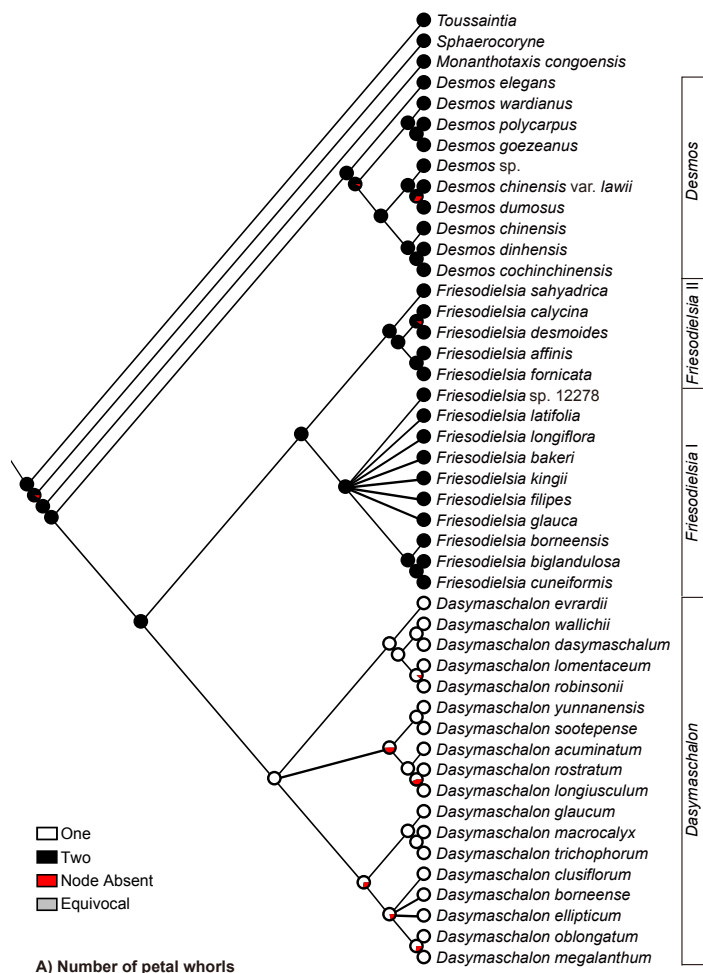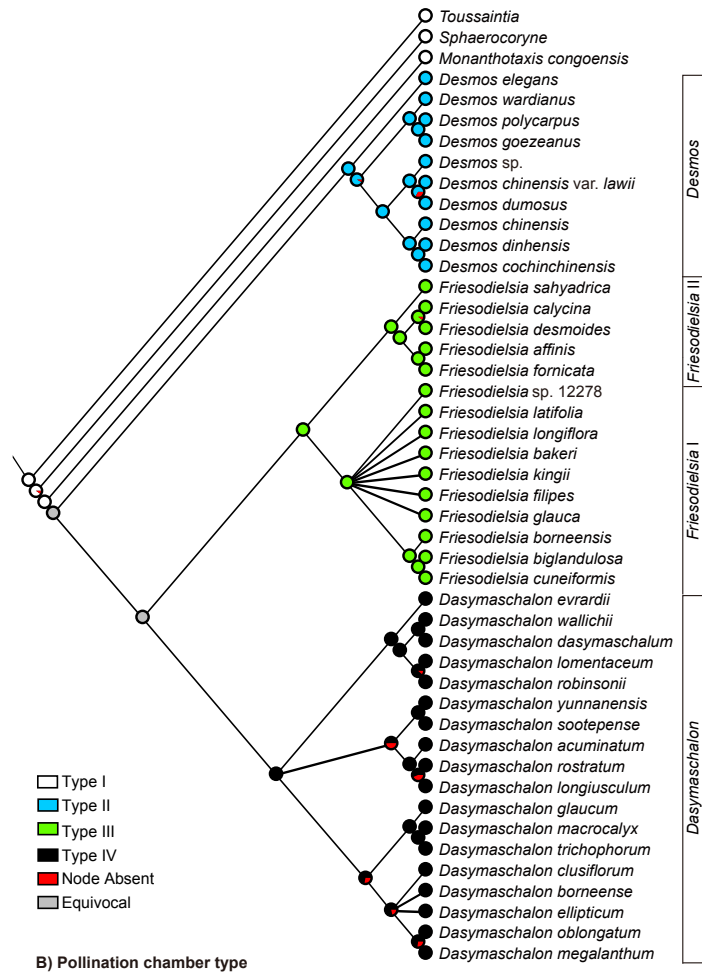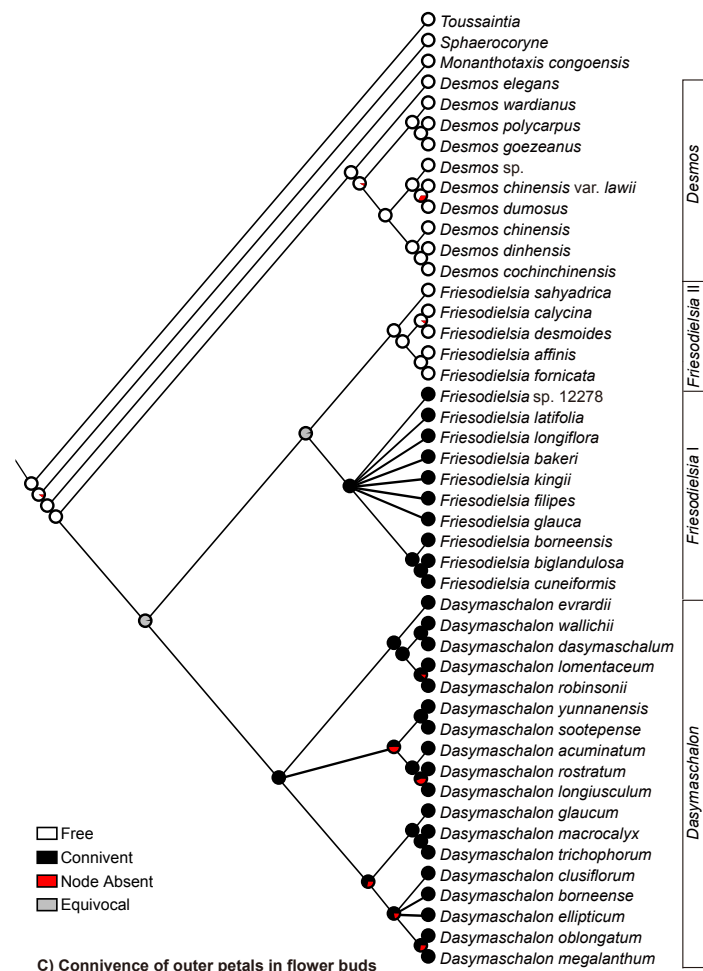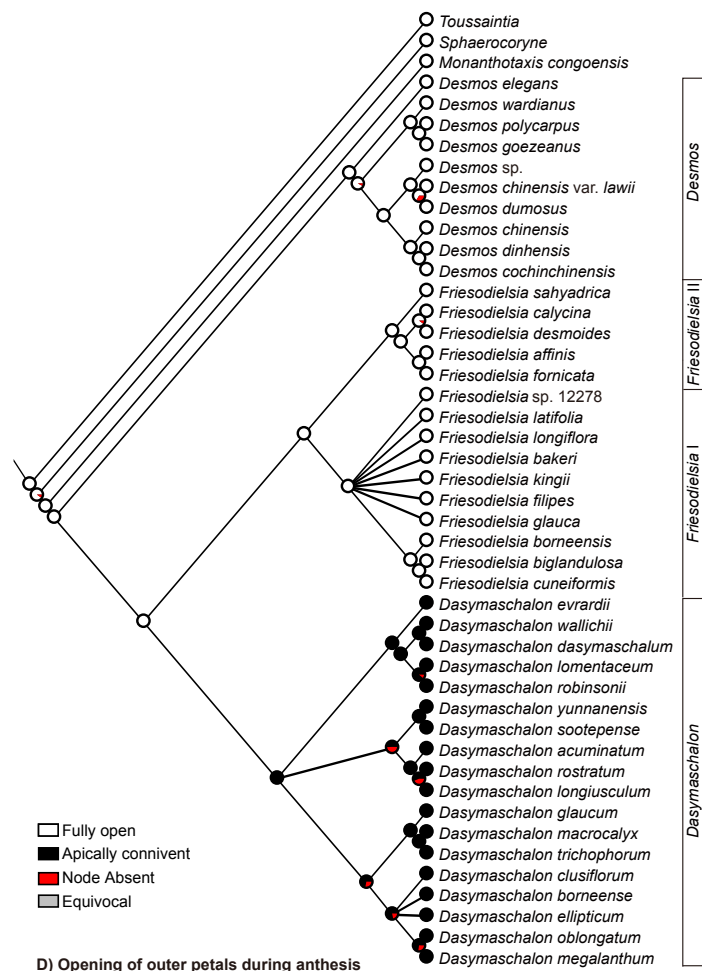

Supplement: FIGURE S1 — Parsimony ancestral character reconstructions for the Desmos–Dasymaschalon–Friesodielsia clade. (A) Number of petal whorls. (B) Pollination chamber type. (C) Connivence of outer petals in flower buds. (D) Opening of outer petals during anthesis. Character reconstructions across 10,000 Bayesian input trees are summarized and mapped on the 50% majority-rule consensus tree based on concatenated dataset from seven chloroplast and nuclear DNA regions. Pie charts at each node show the MP mapping results for the ancestral state and the percentage of node absence in the input trees. [file Image_1.PDF]
