# Supplementary material for: Organ Homologies and Perianth Evolution in the Dasymaschalon Alliance (Annonaceae): Inner Petal Loss and Its Functional Consequences
Source: Front Plant Sci. 2018 Feb 20;9:174. doi: 10.3389/fpls.2018.00174 (PMC5826315; doi:10.3389/fpls.2018.00174)
Supplement: TABLE S1 — Taxon-character data matrix used for ancestral character-state reconstructions. (i) number of petal whorls: 0 = one whorl; 1 = two whorls; (ii) pollination chamber type: 0 = pollination chamber absent, with petals spreading or loosely coherent; 1 = partially closed pollination chamber formed by basally constricted petals, with an apical aperture and three basal apertures; 2 = tightly closed pollination chamber formed by connivent inner petals, with three basal apertures periodically blocked by outer petals; 3 = tightly closed pollination chamber formed by connivent outer petals; (iii) connivence of outer petals when immature: 0 = free; 1 = connivent; (iv) opening of outer petals during anthesis: 0 = becoming fully open; 1 = remaining apically connivent. [file Table_1.PDF]

**TABLE S1. Taxon-character data matrix used for ancestral character-state reconstructions.** (i) number of petal whorls: 0 = one whorl; 1 = two whorls; (ii) pollination chamber type: 0 = pollination chamber absent, with petals spreading or loosely coherent; 1 = partially closed pollination chamber formed by basally constricted petals, with an apical aperture and three basal apertures; 2 = tightly closed pollination chamber formed by connivent inner petals, with three basal apertures periodically blocked by outer petals; 3 = tightly closed pollination chamber formed by connivent outer petals; (iii) connivence of outer petals when immature: 0 = free; 1 = connivent; (iv) opening of outer petals during anthesis: 0 = becoming fully open; 1 = remaining apically connivent.

| Taxon | Characters | | | |
|---|---|---|---|---|
| | i | ii | iii | iv |
| *Dasymaschalon acuminatum* | 0 | 3 | 1 | 1 |
| *Dasymaschalon borneense* | 0 | 3 | 1 | 1 |
| *Dasymaschalon clusiflorum* | 0 | 3 | 1 | 1 |
| *Dasymaschalon dasymaschalum* | 0 | 3 | 1 | 1 |
| *Dasymaschalon ellipticum* | 0 | 3 | 1 | 1 |
| *Dasymaschalon evrardii* | 0 | 3 | 1 | 1 |
| *Dasymaschalon glaucum* | 0 | 3 | 1 | 1 |
| *Dasymaschalon lomentaceum* | 0 | 3 | 1 | 1 |
| *Dasymaschalon longiusculum* | 0 | 3 | 1 | 1 |
| *Dasymaschalon macrocalyx* | 0 | 3 | 1 | 1 |
| *Dasymaschalon megalanthum* | 0 | 3 | 1 | 1 |
| *Dasymaschalon oblongatum* | 0 | 3 | 1 | 1 |
| *Dasymaschalon yunnanensis* | 0 | 3 | 1 | 1 |
| *Dasymaschalon robinsonii* | 0 | 3 | 1 | 1 |
| *Dasymaschalon rostratum* | 0 | 3 | 1 | 1 |
| *Dasymaschalon sootepense* | 0 | 3 | 1 | 1 |
| *Dasymaschalon trichophorum* | 0 | 3 | 1 | 1 |
| *Dasymaschalon wallichii* | 0 | 3 | 1 | 1 |
| *Desmos chinensis* | 1 | 1 | 0 | 0 |
| *Desmos chinensis* var. *lawii* | 1 | 1 | 0 | 0 |
| *Desmos cochinchinensis* | 1 | 1 | 0 | 0 |
| *Desmos dinhensis* | 1 | 1 | 0 | 0 |
| *Desmos dumosus* | 1 | 1 | 0 | 0 |
| *Desmos elegans* | 1 | 1 | 0 | 0 |
| *Desmos goezeanus* | 1 | 1 | 0 | 0 |
| *Desmos polycarpus* | 1 | 1 | 0 | 0 |
| *Desmos* sp. | 1 | 1 | 0 | 0 |
| *Desmos wardianus* | 1 | 1 | 0 | 0 |
| *Friesodielsia affinis* | 1 | 2 | 0 | 0 |
| *Friesodielsia bakeri* | 1 | 2 | 1 | 0 |
| *Friesodielsia biglandulosa* | 1 | 2 | 1 | 0 |
| *Friesodielsia borneensis* | 1 | 2 | 1 | 0 |
| *Friesodielsia calycina* | 1 | 2 | 0 | 0 |
| *Friesodielsia cuneiformis* | 1 | 2 | 1 | 0 |
| *Friesodielsia desmoides* | 1 | 2 | 0 | 0 |
| *Friesodielsia filipes* | 1 | 2 | 1 | 0 |
| *Friesodielsia fornicata* | 1 | 2 | 0 | 0 |
| *Friesodielsia glauca* | 1 | 2 | 1 | 0 |

| | | | | |
|---|---|---|---|---|
| *Friesodielsia kingii* | 1 | 2 | 1 | 0 |
| *Friesodielsia latifolia* | 1 | 2 | 1 | 0 |
| *Friesodielsia longiflora* | 1 | 2 | 1 | 0 |
| *Friesodielsia sahyadrica* | 1 | 2 | 0 | 0 |
| *Friesodielsia* sp. 12278 | 1 | 2 | 1 | 0 |
| *Monanthotaxis congoensis* | 1 | 0 | 0 | 0 |
| *Sphaerocoryne affinis* | 1 | 0 | 0 | 0 |
| *Toussaintia orientalis* | 1 | 0 | 0 | 0 |
